# Protein-Mediated Precipitation of Calcium Carbonate

**DOI:** 10.3390/ma9110944

**Published:** 2016-11-22

**Authors:** Izabela Polowczyk, Anna Bastrzyk, Marta Fiedot

**Affiliations:** 1 Faculty of Chemistry, Wroclaw University of Science and Technology, Norwida 4/6, 50-373 Wroclaw, Poland; anna.bastrzyk@pwr.edu.pl; 2 Faculty of Microsystem Electronics and Photonics, Wroclaw University of Science and Technology, Janiszewskiego 11/17, 50-372 Wroclaw, Poland; marta.fiedot@pwr.edu.pl

**Keywords:** lysozyme, ovalbumin, mixture of proteins, biomineralization, calcite, Turbiscan

## Abstract

Calcium carbonate is an important component in exoskeletons of many organisms. The synthesis of calcium carbonate was performed by mixing dimethyl carbonate and an aqueous solution of calcium chloride dihydrate. The precipitation product was characterized by means of scanning electron microscopy (SEM), transmission electron microscopy (TEM), X-ray diffraction (XRD), and Fourier transform infrared spectroscopy (FTIR) measurements. In addition, the turbidity of the reaction solution was acquired to monitor the kinetics of the calcium carbonate structure’s growth in the investigated system. In this study, samples of CaCO_3_ particles obtained with individual proteins, such as ovalbumin, lysozyme, and a mixture of the proteins, were characterized and compared with a control sample, i.e., synthesized without proteins. The obtained data indicated that the addition of ovalbumin to the reaction changed the morphology of crystals from rhombohedral to ‘stack-like’ structures. Lysozyme, however, did not affect the morphology of calcium carbonate, yet the presence of the protein mixture led to the creation of more complex composites in which the calcium carbonate crystals were constructed in protein matrices formed by the ovalbumin-lysozyme interaction. It was also observed that in the protein mixture, ovalbumin has a major influence on the CaCO_3_ formation through a strong interaction with calcium ions, which leads to the coalescence and creation of a steric barrier reducing particle growth. The authors proposed a mechanism of calcium carbonate grain growth in the presence of both proteins, taking into account the interaction of calcium ions with the protein.

## 1. Introduction

Calcium carbonate is an important biogenic mineral used by nature as an inorganic component in tissues and exoskeletons of many mineralizing organisms [1,2,3]. These biocompatible inorganic microparticles have become an active area of research due to their exquisite nanostructures, superior mechanical properties, and unique biological functions, such as support, protection, sensing, storage, and homeostasis [4,5].

Biogenic minerals, calcium carbonate in particular, are routinely used as templates for the fabrication of hollow multilayered microcapsules [6]. This fascination with biogenic minerals results in a need to control the factors associated with the formation of these materials, such as size distribution, morphology, and stability of templates [4,6]. Typically, calcium carbonate exists in many forms, including hydrates (amorphous calcium carbonates; ACC) and anhydrates (calcite, vaterite, and aragonite) [7,8]. Among these forms, calcite is the most thermodynamically stable, and most of the calcium carbonate in nature occurs in this form. In contrast, vaterite is the least stable of the three structures of anhydrous crystalline CaCO_3_, but it plays an important role in calcium carbonate formation [9]. In addition, vaterite may be potentially useful in a wide range of biomedical and industrial applications due to its higher solubility, dispersion, and specific surface area compared to calcite and aragonite [4,10,11].

In living organisms, the process of cell-controlled biomineralization leads to the synthesis of extremely stable and unusual three-dimensional calcium carbonate microparticles utilized by host organisms as skeletons and protective shells [6,7]. Biogenic CaCO_3_ minerals are composed of inorganic CaCO_3_ and a minor organic matrix (proteins and polysaccharides) [12,13,14]. In vitro precipitation tests indicated that these macromolecules play a crucial role in the formation of biominerals not only by providing a structural framework, but also by regulating the entire dynamic process including the nucleation site, direction of orientation, growth, and crystal assembly [4,15,16,17,18,19]. Several mechanisms have been proposed to explain the interactions between protein and mineral surfaces, including a combination of electrostatic and stereochemical interactions, as well as geometrical matching [3,20,21,22]. The dominance of one type of interaction is dependent on protein characteristics (i.e., size, conformation, surface charge, type of functional groups), biomineral composition, and solution properties [18].

Avian eggshells are representative nanocomposites consisting of natural biopolymers and calcium carbonate with highly ordered structures and biological functions [4,12,19]. Calcium carbonate exists in the form of calcite in the mammillary knob and palisade layers of an eggshell. The shape, size, and orientation of the calcite crystals in both layers are well regulated by organic matrix components [12]. The organic matrix participating in the calcification of an eggshell consists of proteins, such as osteopontine and clusterin, egg white proteins, such as ovalbumin, lysozyme, and ovotransferrin, and other macromolecules [23]. In addition, during the initial stage of shell formation, ovalbumin, ovotransferrin, and lysozyme are present in the environment where the calcification of the shell occurs [24]. In recent years, based on fact that the biomineralized materials often contain proteins that are rich in glutamic or aspartic acid residues, various single proteins have been developed to control the mineralization process of calcium carbonate in a solution [23,24,25,26]. Due to the high complexity of the constituents of the organic matrix, the eggshell formation mechanism and the contribution of each constituent remain poorly understood, and more investigation is required using model systems where mixtures of macromolecules are present. The application of a lysozyme and ovalbumin mixture in the reaction solution is the novel aspect of this research. These macromolecules can interact with each other in an aqueous solution resulting in the formation of lysozyme-ovalbumin aggregates, which can lead to the formation of complex structures of calcium carbonate clusters. In this work, it was observed that calcium carbonate particles are incorporated into protein matrices creating organic-inorganic solid hybrids.

## 2. Results and Discussion

### 2.1. Kinetics of Calcium Carbonate Crystals Formation

Understanding the mechanism of biomaterials formation in the presence of biomolecules is important to mimic the synthesis of the inorganic materials with unique properties and morphology, which are present in nature. An attempt to fabricate the organic/inorganic materials outside biological organisms will lead to the manufacturing of new composites with potential uses in many fields. The research described in this paper can provide a more valuable insight into the role of ovalbumin and lysozyme in the formation of calcium carbonate composites. These proteins, at the same time, are present in fluid during the calcification process of a hen eggshell. More thorough studies are still needed to evaluate how not only individual proteins, but also a mixture of these proteins, control the formation of biomaterials.

To prevent fast precipitation of calcium carbonate in an aqueous solution containing Ca^2+^ ions, dimethyl carbonate (DMC) was used as a source of CO_2_ [27]. During the preparation of calcium carbonate, several chemical reactions may occur (see Reactions (1)–(6)). In the first stage, dissociation reaction of CaCl_2_ takes place (Reaction (1)). Hydrolysis of DMC in the calcium chloride solution does not occur without NaOH [27]. When the protein is added to a solution containing DMC and calcium ions, calcium-protein complexes (Reaction (2)) can be formed [28]. If NaOH is added to a solution containing DMC molecules, calcium ions and the protein, two competitive reactions can occur; i.e., calcium hydroxide formation preceded by the calcium-protein complex decomposition (Reaction (3)) and DMC hydrolysis (Reaction (4)) [27]. The rate of the first reaction depends on the complex decomposition rate. If the interaction energy is low, the decomposition rate is higher than the rate of Reaction (4) and at first calcium hydroxide is formed, which reacts immediately with the carbonate ions (Reaction (5)). If the rate of Reaction (4) is higher than the complex decomposition rate, direct calcium carbonate formation occurs (Reaction (6)). As in this study the solubility product of calcium hydroxide has not been exceeded, it is possible to assume that the Reactions (3) and (5) are negligible.

(1) $$CaCl_{2}\to Ca^{2+}+2Cl^{-}$$

(2) $${Ca}^{2+}+ovalbumin\to \left[ovalbumin-Ca\right]$$

(3) $$Ca^{2+}+2OH^{-}\to Ca{\left(OH\right)}_{2}$$

(4) $${\left(CH_{3}O\right)}_{2}CO+2OH^{-}\to CO_{3}^{2-}+2CH_{3}OH$$

(5) $$Ca{\left(OH\right)}_{2}+CO_{3}^{2-}\to CaCO_{3}+2OH^{-}$$

(6) $$CO_{3}^{2-}+Ca^{2+}\to CaCO_{3}$$

**Figure 1:**
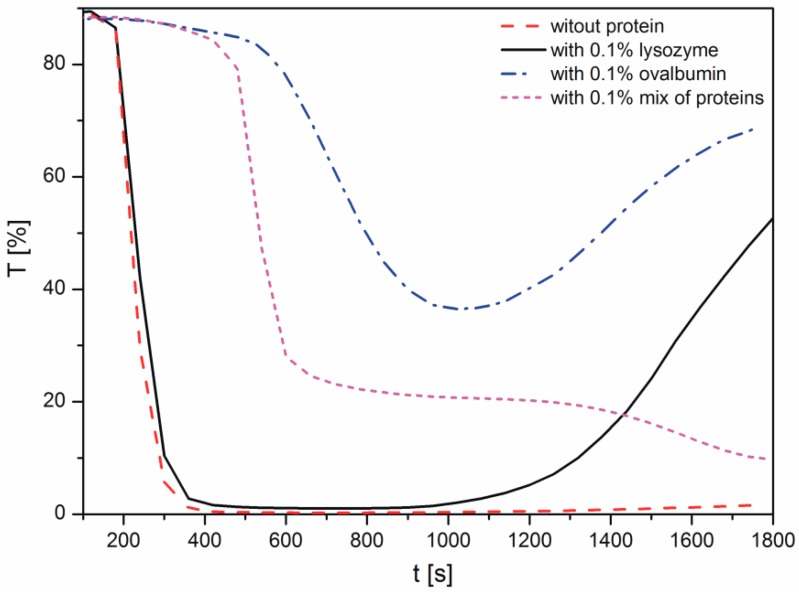
Transmission curves observed during precipitation of calcium carbonates.

Changes in the light transmission in the reaction solution during calcium carbonate formation in the presence of proteins are shown in Figure 1. The samples were analyzed immediately after mixing of the reactants to observe the crystallization process. During the measurements conducted with the Turbiscan apparatus, the reaction mixture was not stirred. In the course of measurement, the transmission detector receives the light that passes through the sample [29]. Therefore, when the transmission is higher, there are fewer particles present in the suspension. The decrease in the transmission, i.e., the increase in the sample turbidity, corresponds to progressive precipitation of calcium carbonate particles. A resurgence in transmission results from the growth in calcium carbonate size and particle sedimentation.

As shown in Figure 1, in the sample without proteins, the transmission decreased rapidly within 200 s, indicating the precipitation of CaCO_3_. The addition of lysozyme to the reaction solution only slightly influenced the transmission profile and thus the precipitation rate. These data indicated that the protein has only a small effect on calcium carbonate growth, compared with the CaCO_3_ sample without a protein. After approximately 17 min, an increase in the transmission is the result of sedimentation of larger aggregates of the precipitated particles. This effect may be due to the H-bonding between the arginine residues of lysozyme and the neutral calcium carbonate species [12].

When ovalbumin was present, the transmission values were higher than those in the sample without proteins, due to a lower concentration of crystals in suspension. Therefore, based on this observation, the presence of ovalbumin retarded the crystallization process. The decrease in the transmission was observed within 600 s, however, it was not so sharp compared with the lysozyme and control samples. In addition, it is important to note that in the investigated system, ovalbumin and calcium carbonates created aggregates. After approximately 17 min, sedimentation of the particles in the presence of this protein was observed due to a large size of the ovalbumin-calcium carbonate grains.

The presence of an ovalbumin-lysozyme mixture resulted in a shift in the transmission profile. An abrupt increase in the turbidity of the sample appeared at 500 s. Nonetheless, in both cases, the transmission of the sample has not disappeared completely but dropped to the level of 35% and 20% for ovalbumin and mix of proteins, respectively.

The authors supposed that the area of the diagram, where high transmission at the beginning of the process is observed, is correlated with reactions occurring in the solution (Reactions (1) and (2)). The longer the step is, the higher the decomposition energies of intermediate products of the reaction are (Reaction (2)). Subsequently, a sudden decrease of transmission curves is distinctly noticeable. As it was noted, a fall in the transmission value is caused by precipitation of CaCO_3_ which is due to further Reactions (4)–(6) (Figure 1). For this reason, it could be concluded that ovalbumin creates a strong complex with calcium, which has not been observed in the case of lysozyme. Additionally, we can suppose that calcium carbonate particles with adsorbed proteins showed different properties of the mineral surface. Depending on the adsorbed protein, the surface can have various physicochemical properties and it can cause changes in the profile of the transmission curve during crystallization.

### 2.2. SEM Analysis of the Calcium Carbonate Crystals

**Figure 2:**
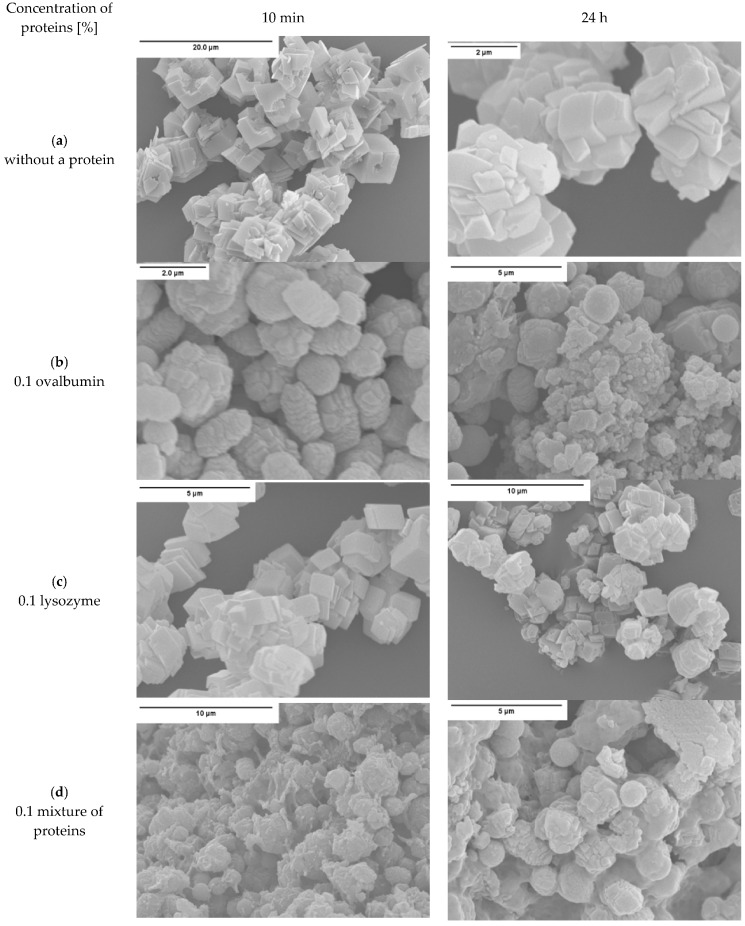
SEM images of CaCO_3_ obtained in the presence of: (**a**) no protein; (**b**) ovalbumin; (**c**) lysozyme; and (**d**) mixture of proteins.

**Figure 3:**
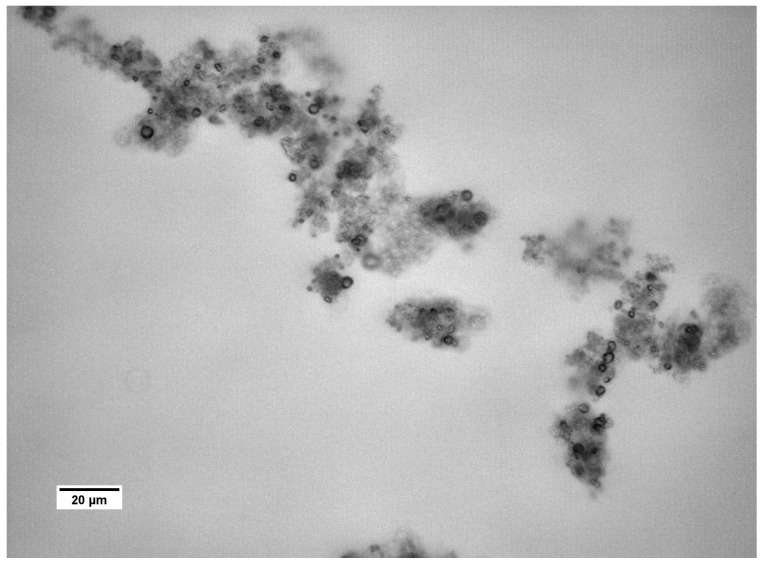
Micrograph of organic-inorganic agglomerates obtained in the presence of a protein mixture (0.1% of albumin and lysozyme, 24 h).

The morphologies of the CaCO_3_ particles precipitated in the absence and in the presence of ovalbumin and lysozyme are shown in Figure 2 and Figure 3.

Based on the analysis of scanning electron microscopy (SEM) images shown in Figure 2a, the calcium carbonates formed without any additives consisted of rhombohedral calcite crystals with smooth surfaces after 10 min and 24 h of precipitation. Particles precipitated in the presence of ovalbumin are made up of numerous thin layers and do not form well-defined crystals (Figure 2b). Ovalbumin changed the morphology of the crystals. At a neutral pH, this biomacromolecule possesses a large number of negatively-charged carboxyl groups [26]. According to literature data, the protein macromolecules formed a framework at the solution surface, and the calcium ions were bound to these chains via the carboxyl groups [26,30,31]. The size of particles synthesized in the presence of ovalbumin is not dependent on the duration of the process. In the presence of lysozyme well-defined crystals were formed, just like without the protein (Figure 2c). Microstructure of calcium carbonate grain precipitated in the presence of both proteins is identical to the grains formed in the presence of ovalbumin (Figure 2d). This suggests that ovalbumin has a decisive influence on the kinetics of carbonate formation.

The data shown in Figure 2c indicates that the addition of lysozyme had a weak influence on the morphological rhombohedral calcite. These data are in a good agreement with previous results [32]. This is due to the lysozyme possessing 18 positively-charged surface amino acids [12]. In this reaction system, no attractive electrostatic interaction which can lead to significant changes in the morphology of crystal lattice occurs between the calcium ions, the surface planes of CaCO_3_ and the biopolymer macromolecule.

The structure of calcium carbonate agglomerates formed in the protein mixture matrix can be seen in Figure 3. The addition of both proteins to the reaction mixture resulted in the formation of complex composite agglomerates (Figure 2d and Figure 3). Based on the results in Figure 3, it can be concluded that the presence of a mixture of ovalbumin and lysozyme during the crystallization process led to the creation of calcium carbonate, which is distributed within protein structures. SEM image analysis (Figure 2d) revealed that at a biopolymer mixture concentration of 0.1%, the shape of the crystals corresponded to spherules and ‘stack-like’ structures. After drying, the crystals agglomerated and created a tightly-packed matrix, where the particles of calcium carbonate are linked with protein chains.

**Figure 4:**
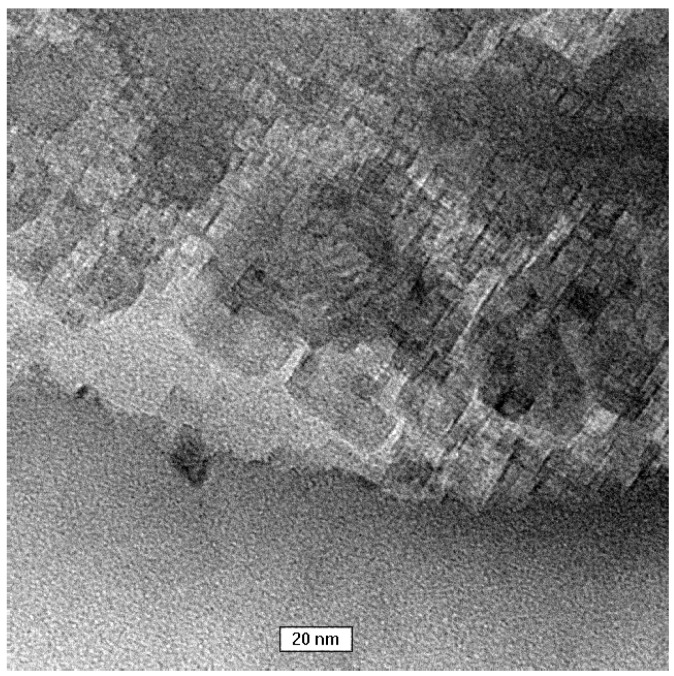
TEM image of CaCO_3_ precipitated without protein, after 1 min.

A transmission electron microscopy (TEM) image of the control sample is shown in Figure 4. After 1 min the reaction mixture was poured into correspondingly higher volumes of isopropanol to slow down the crystallization process at a very early stage. Consequently, smaller particles were created. The obtained results showed that the crystallized particles have a spherical shape and are made of nanoparticles. Literature data suggest that this structure is typical of vaterite [33]. This supports the assertion that vaterite is a transient product of calcite synthesis.

### 2.3. XRD Analysis of the Calcium Carbonate Crystals

**Figure 5:**
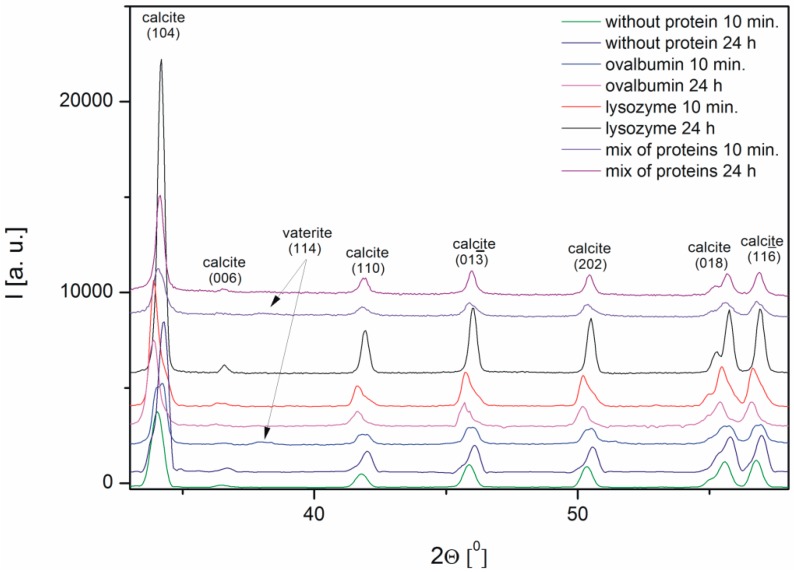
XRD diffraction patterns of the crystal structure of the calcium carbonates precipitated in the absence and in the presence of proteins (0.1%).

The characterization of the calcium carbonate crystals was carried out using powder X-ray diffraction. Figure 5 shows the X-ray diffraction patterns of CaCO_3_ obtained in the absence and in the presence of proteins, such as ovalbumin, lysozyme, and a mixture of both proteins. The X-ray diffraction (XRD) data indicated that calcite was the precipitate obtained after 24 h both in the absence and in the presence of the proteins. The peak characteristic of calcite at 2Θ = 34.04° corresponded to the (104) crystallographic plane of the calcite observed in all the samples [34]. The analysis of the XRD patterns of calcium carbonate precipitate after 10 min revealed that small peaks corresponding to vaterite appeared in the samples with 0.1% of ovalbumin and the protein mixture.

**Figure 6:**
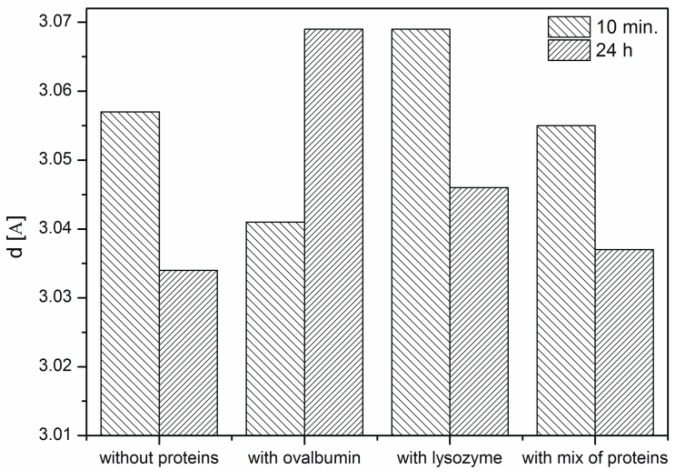
Interplanar spacing d of CaCO_3_ samples after 10 min and 24 h. Protein concentration—0.1%.

Based on the obtained XRD data the interplanar distances corresponding to the (104) crystallographic plane were calculated using Bragg’s equation (Figure 6). It is known from the literature that the d-spacing of this plane is 3.05 [10,13]. In the samples with lysozyme and without protein the ordering of the structure is observed, i.e., the interplanar distance is changing. An increasing interplanar spacing is observed in the samples with ovalbumin and the protein mixture. At the protein mixture concentration of 0.1% competition between both proteins is confirmed. We suspect that the interaction between ovalbumin and lysozyme can partially block the interaction between Ca^2+^ ions and ovalbumin. Lysozyme does not block the crystallization process compared to ovalbumin, which shows weak interaction of lysozyme with Ca^2+^ ions. In contrast, ovalbumin is incorporated into the crystallite structure.

The average crystallite size and microstrains in CaCO_3_ were determined by means of the Williamson-Hall (W-H) method [35]:

(7) $$\beta \cos\left(\varphi \right)=\frac{k\lambda }{D}+4\varepsilon \sin\left(\varphi \right)$$

where:

*β*—half-width of the (FWHM) peak (rad),
*φ*—the angle of the highlight for the given band interference (rad),
k—the Scherrer constant (the 0.9 value),
*λ*—the wavelength of the X-ray beam (nm),
D—the average crystallite size (nm),
*ε*—microstrain.

**Figure 7:**
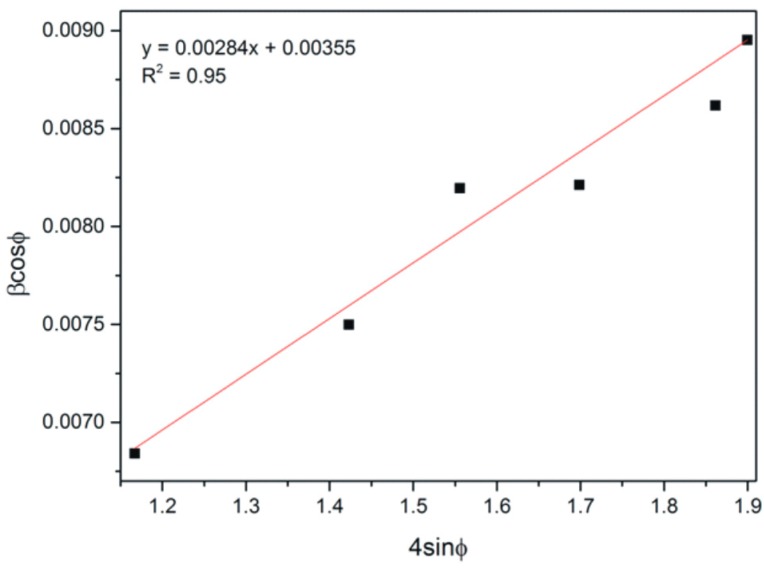
The W-H analysis of CaCO_3_ precipitated with lysozyme (0.1%) after 10 min.

This equation takes into account uniform strain in all crystallographic directions, thus assuming the isotropic nature of the analyzed crystal by plotting of the *β*cos(*φ*) = f (4sin*φ*) curve for diffraction peaks connected with calcite. The W-H analysis of CaCO_3_ with lysozyme after 10 min was demonstrated (Figure 7).

**Figure 8:**
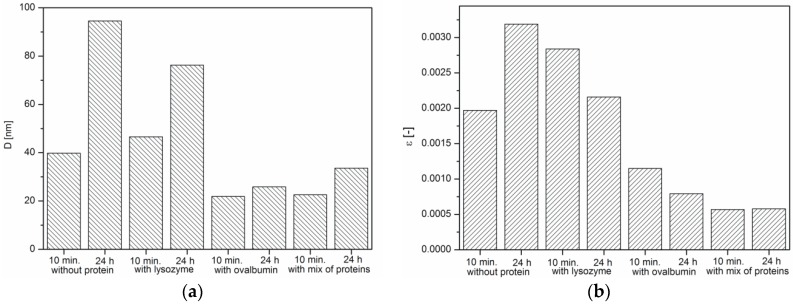
Changes in: (**a**) crystallite size; (**b**) microstrains. Protein concentration: 0.1%.

It was indicated that the crystallite size of all samples increases within 24 h, which is characteristic of crystallization process (Figure 8a). It was also observed that microstrains in crystals decreased, except the CaCO_3_ sample obtained without the protein (Figure 8b). The presence of proteins affects the growth of calcium carbonate grains, but depending on the protein type, to a varying extent. According to the literature, a decrease in the microstrains is a result of the hardening of crystals [36], as well as the incorporation of the organic inclusions within the crystals [37]. On the other hand, microstrain increases because of the formation of crystal-crystal interfaces, which leads to partial clamping of individual crystals by the surrounding particles. The boundaries between the primary crystals were argued to be the source of the microstrain [38]. Based on SEM images of precipitated calcium carbonate (Figure 2b,d), a decreasing crystal boundary density resulted in decreased microstrains. For this reason, CaCO_3_ samples precipitated without the protein and with lysozyme (Figure 2a,c) showed larger crystallite size and, thus, higher microstrain values.

### 2.4. FTIR Analysis of Calcium Carbonate Crystals

**Figure 9:**
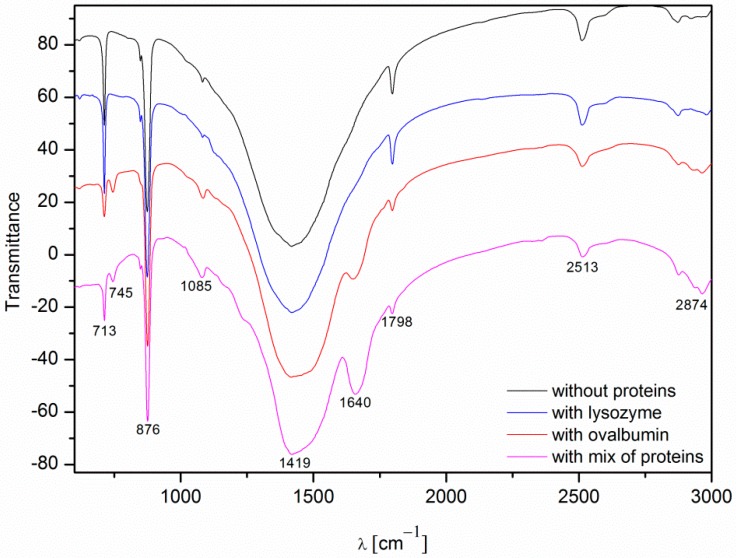
FTIR spectra of the calcium carbonate crystals obtained in the absence and in the presence of proteins (0.1%, after 10 min).

Fourier transform infrared spectroscopy (FTIR) spectroscopy was used as a secondary characterization technique to identify various polymorphs present in the crystal. The FTIR spectra of the calcium carbonate crystals obtained in the absence and in the presence of the proteins (0.1%) after 10 min of precipitation are shown in Figure 9. The band assignment of the FTIR vibrations of the calcium carbonate particles are presented in Table 1.

The spectra of calcium carbonate contained the peaks characteristic of calcite at ~1420, ~874, and ~712 cm^−1^ [4,10,39,40]. According to the data shown in Figure 5, the only crystalline form of CaCO_3_ obtained without proteins and with 0.1% of lysozyme was calcite. For the samples obtained with 0.1% of ovalbumin and the protein mixture (Figure 9), new peaks located at ~745 cm^−1^ appeared [4,10]. These data suggested that the addition of ovalbumin resulted in the presence of both the vaterite and calcite phases after 10 min precipitation. In addition, when comparing all of the data in Figure 9, the additional peak located at ~1640 cm^−1^ was present in the ovalbumin-CaCO_3_ and ovalbumin-CaCO_3_-lysozyme systems. This peak was assigned to the I amide band, which is characteristic of proteins [41]. In nature, calcium carbonate can be found in the form of a composite material with various macromolecules [20,22]. Therefore, ovalbumin may create aggregates with calcium carbonate due to a supramolecular interaction between the protein and calcium carbonate leading to formation of an ovalbumin-Ca complex [30]. In the solution, the secondary structure of the protein is affected in the course of the reaction. Some of the α-helices are stretched and changed into β-sheets, resulting in protein denaturation [30,41]. A similar behavior was observed by Ichikawa and co-workers [42], who obtained an inorganic-organic complex that consisted of calcium carbonate and poly(l-aspartate). Hu and co-workers [13] reported that calcium carbonate-egg white aggregates were formed during precipitation. In addition, Zhao and co-workers [43] demonstrated that calcium carbonate crystals interacted with the sericin protein to form CaCO_3_/protein clusters, which was confirmed by FTIR and thermogravimetric analysis. Zhu and co-workers [44] suggested that a copolymer (i.e., *β*-cyclodextrin-b-poly(l-glutamic acid)) can be incorporated into calcium carbonate crystals. In our experiments, this phenomenon was observed in the presence of ovalbumin and the protein mixture. The isoelectric point of ovalbumin and lysozyme is 4.8 and 11.0, respectively [13,45]. In the current study, the pH of the solution during precipitation was approximately 8.0. Under these conditions, lysozyme has a positive net charge and weak affinity for calcium cations. Therefore, lysozyme may weakly act as a nucleation center and increase the rate of precipitation [24]. According to the literature, the zeta potential of freshly-precipitated pure calcium carbonate crystals is positive [46]. Therefore, the electrostatic reaction of lysozyme with the surface of CaCO_3_ crystals could be repulsive under these conditions. Similar results were reported by Hernández-Hernández and co-workers [12], who observed that the only interaction under these conditions was the H-bonding between arginine residues of lysozyme and the step edges of neutral species of calcium carbonates. However, the addition of ovalbumin delayed the crystallization process. The pH of the reaction solution results in a negative net surface charge on ovalbumin due to charged carboxyl groups [26]. Capture and complexation of Ca^2+^ by ovalbumin influences the nucleation and favors the formation of surfaces covered with chemisorbed protein [30]. This protein may act as a chelating agent and an inhibitor of nucleation during CaCO_3_ precipitation [4,12].

### 2.5. Mechanism of CaCO_3_ Crystallization

**Figure 10:**
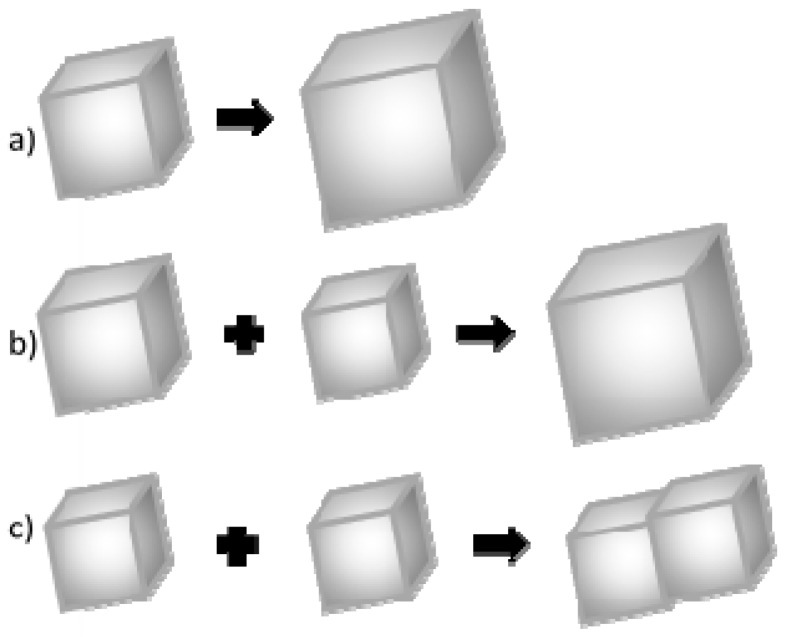
Mechanisms of calcium carbonate crystal growth: (**a**) attachment of new ions to the existing crystallite; (**b**) Ostwald’s recrystallization; and (**c**) coalescence.

It is assumed that the crystal grain growth may happen once its critical size has been exceeded. This growth occurs according to one of the three mechanisms: (Figure 10) [47,48]:

(a) Association of more atoms/ions to the existing crystal;
(b) Ostwald ripening, i.e., dissolution of smaller crystal grains and growth of larger ones;
(c) Coalescence.

In low concentration solutions, the association of calcium and carbonate ions to the surface of existing crystal grain is usually a dominant process of crystal growth. The second most common process is Oswald’s ripening [49]. This is based on a dissolution of smaller calcium carbonate particles and an increase in the size of larger ones. The third process—coalescence—is a concrescence of nanocrystallites and the formation of a larger one, and then its growth as a whole. In a typical coalescence process, an alignment of the crystal lattice is retained. Coalescence is a fairly common mechanism of crystallites growth. It proceeds intensively immediately after a rapid nucleation process when the Brownian motion energy of nanocrystals is too low to compensate for the force of van der Waals interaction between particles in close proximity.

Based on the obtained results, the authors concluded that the mechanism governing calcium carbonate crystals growth in this case depends on the composition of solution. In the solution without protein, the association of calcium and carbonate ions as a consequence of DMC hydrolysis is a prevailing process in the formation of calcium carbonate particles (Figure 10a). Due to the physicochemical properties of the precipitating agent (DMC), the process of crystallites growth is retarded because it depends on the DMC hydrolysis rate [50]. TEM results have shown that in this solution vaterite particles were formed at the initial stage and then transformed into calcite (Figure 4). Additionally, in the solution without protein the crystallite growth is a result of Oswald’s recrystallization, as reflected in the particle size distribution. In this case, an increase in the microstrains in the crystal lattice is observed after 24 h, which is a result of a vaterite transformation into calcite polymorph. Furthermore, as demonstrated in SEM images (Figure 2a,c), calcite particles are formed with stacked crystals confirming the coalescence growth mechanism in the presence of lysozyme and without any additives.

The addition of protein to the solution changes its viscosity, which affects the mobility of ions. As a result, the diffusion rate of carbonate ions and CaCO_3_ nanocrystallites should decrease in the presence of both proteins. Furthermore, proteins can interact with calcium ions and form complexes [51].

In the solution containing lysozyme, both the microstructure and the mean size of crystallites are the same as in the case of calcium carbonate precipitated without the protein. Therefore, it must be assumed that the influence of this protein on the mobility of ions is minor. Additionally, this protein does not create strong bonds with calcium ions. The growth of crystallites is a result of both Oswald’s recrystallization and coalescence, as in the case of no protein presence.

**Figure 11:**
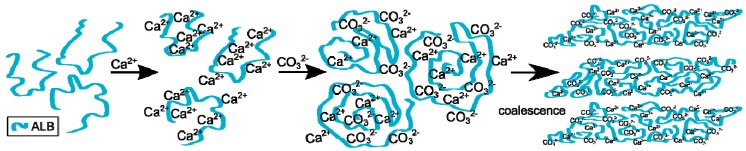
Scheme of the process of calcium carbonate formation in the presence of ovalbumin.

The process of calcium carbonate formation is different in the presence of ovalbumin. The obtained SEM and XRD results (Figure 2 and Figure 5) showed considerable, visible changes in the morphology of the calcium carbonate crystals in the presence of this protein. The CaCO_3_ particles obtained in this case consisted of irregular ‘cone-like’ crystals. No rhombohedral crystals were present in the precipitate. Similar results were observed by Hu and co-workers [13] where this type of morphology was referred to as a ‘stack-like’ structure that consists of many thin sheets. Zheng and co-workers [1] obtained calcium carbonate structures in the presence of egg white, and these structures consisted of many thin sheets. Based on these results, the multilayered structure of the organic/inorganic matrix in egg shells can result from the presence of ovalbumin in avian organisms. This phenomenon led to a locally high concentration of calcium ions inside the framework and induced nucleation [26]. At an early stage of the reaction, amorphous calcium carbonate is formed [1,13]. Carboxyl groups in the protein can bind to calcium ions, as well as the specific crystal plane of calcium carbonate [13,24]. This strong interaction disturbs the formation of typical calcite rhombohedra. The authors believe that ovalbumin can bind calcium ions and create ‘stack-like’ structure (Figure 11).

Next, the hydrolysis of DMC occurs through the action of sodium hydroxide and carbonate ions are formed. These ions break up the Ca-ovalbumin complex and calcium carbonate is formed. Since the decomposition of complex requires time, it can be distinctly seen in the transmission curve of the solution. Calcium carbonate particles are formed as a result of coalescence, which is clearly seen from SEM images (Figure 2). However, the coalescence does not lead to the concrescence of nanocrystallites with a complete alignment of the crystal lattice. Due to the properties of this protein, it should be concluded that ovalbumin strongly binds calcium ions, which contributes to the coalescence and, in addition, forms a steric barrier preventing particle growth.

In the presence of the protein mixture, ovalbumin has a decisive influence on the calcium carbonate formation, whereas the lysozyme has an indirect impact. For calcium carbonate precipitated in such conditions, the average crystallite size, as well as microstrain (Figure 8a,b) were found to be at their lowest. Additionally, transmission changes during the precipitation process were affected by the presence of the mixture of proteins, as can be seen in Figure 1. In addition, based on the analysis of the SEM images (Figure 2d), more complex structures were obtained when the lysozyme-ovalbumin mixture was employed. At low ionic strength and pH 8.0, the net charges of lysozyme and ovalbumin are positive and negative, respectively [9,30]. According to DLVO theory, under these conditions, the lysozyme-ovalbumin association is possible due to attractive electrostatic interactions [52,53]. Furthermore, under suitable conditions, calcium-induced denaturation can result in self-aggregation of ovalbumin chains [31]. Due to a partial screening of the net charge of proteins by electrolyte ions, hydrophobic interactions between ovalbumin and lysozyme can be involved [52]. Both types of interactions play a crucial role in the formation of organic-inorganic clusters. In such a system, the calcium ions that most likely bind to ovalbumin can be entrapped in the protein matrix due to the ovalbumin-lysozyme interaction. The diffusion of carbonate ions into the matrix can decrease due to an increase in the viscosity of the solution, resulting in the formation of small, spherule calcium carbonate particles located in the protein matrix. Based on these observations, it can be concluded that the addition of the mixture of ovalbumin and lysozyme affects the calcium carbonate structure in a completely different way than the individual protein. TEM measurements of CaCO_3_ particles obtained in the presence of the protein mixture confirm this statement.

## 3. Experimental

### 3.1. Materials

Calcium chloride dihydrate (purity > 99%) (Sigma-Aldrich, Saint Louis, MO, USA), dimethyl carbonate (DMC) (Sigma-Aldrich CHEMIE, Steinheim, Germany), sodium hydroxide standard solution (1.0 M) (POCh, Gliwice, Poland). Lysozyme (Sigma-Aldrich), ovalbumin (EMD Biosciences, San Diego, CA, USA) from chicken egg white. All of the chemicals were of analytical grade and were used as supplied without any further purification. Purified water with the conductivity of less than 1 μS/m was obtained from milli-Q system Pacific 40 (TKA, Niederelbert, Germany).

### 3.2. Methods

The preparation of calcium carbonate was performed according to the method reported by Faatz [27]. The aqueous solution was prepared in a 100-mL glass flask containing 147 mg of CaCl_2_·2H_2_O, 450 mg of DMC and 0.1% (*wt*/*wt*) protein and diluted to 100 mL with water. The reaction was triggered by adding 2.0 mL of 1.0 M NaOH to a stirred reaction mixture. The pH of the solution was about 8.0. The solution was stirred for 4 h and then left to stand under static conditions. After 24 h, the precipitate was removed from the solution by centrifugation. The powder was collected and washed several times with water. The experiments were conducted at an ambient temperature (25 °C). The control sample was performed without protein.

The turbidity of the suspensions during calcium carbonate synthesis was investigated using a Turbiscan Lab^EXPERT^ instrument (Formulaction, L’Union, France). The apparatus can detect and measure small changes in the suspension behavior by means of transmission (T) and backscattering (BS) light collection. The light source is an electroluminescent diode (*λ*_air_ = 880 nm). Two synchronous optical sensors gather light transmitted through the sample and light backscattered by the sample. In the Turbiscan Lab, the optical reading head scans a sample every 40 μm to acquire the transmission and backscattering data. Transmission is used to analyze transparent to turbid dispersions, and backscattering is used to analyze opaque dispersions. In each experiment, 20 mL of the reaction mixture were added to a special glass cell and placed in the Turbiscan apparatus immediately after the sodium hydroxide was added to trigger the reaction. The results were processed using the Turbisoft ver. 1.13 software to obtain the transmission changes as a function of time.

Scanning electron microscopy (SEM) was performed using a JMS-5800LV (JEOL, Akishima, Japan) instrument. The secondary electrons were used to obtain the SEM micrographs. The accelerating electron voltage was 10 kV and the working distance was 10 mm.

Microscopic photos of the calcium carbonate precipitate were recorded in transmission mode with an optical microscope AxioImager M1m (Zeiss, Jena, Germany).

X-ray diffraction (XRD) (Bourevestnik, Sankt Peterburg, Russia) measurements were performed using a DRON 2 diffractometer with Co radiation filtered with Fe. Measurements were carried out by a step recording method with a shift Δ2Θ = 0.05° in the angle range 10°–66° 2Θ. Based on the obtained diffractograms, the interplanar distances were determined using Bragg’s equation for each diffraction pick, characteristic of CaCO_3_. The average crystallite size and the microstrains in CaCO_3_ were determined by means of the Williamson-Hall (W-H) method [35].

To investigate the crystallization mechanism, transmission electron microscopy (TEM) was performed using a TecnaiG2 20X-TWIN instrument (FEI, Hillsboro, OR, USA) for the samples of CaCO_3_ precipitated without protein. In this case, the reaction mixture was poured into excess volume of isopropanol after approximately 1 min to stop the crystallization process at a very early stage.

Fourier transform infrared spectroscopy (FTIR) was carried out using a PE1600 FTIR spectrometer (Perkin Elmer, Waltham, MA, USA). The samples were mixed with KBr powder. The spectra were recorded in reflection mode from 4000 to 400 cm^−1^ at a resolution of 2 cm^−1^.

## 4. Conclusions

In summary, an abundance of biomaterials exist in nature, and these materials typically form composites of inorganic and organic materials in a highly organized manner with fascinating shapes, structures, and excellent properties. In this study, lysozyme, ovalbumin, and a mixture of both proteins, were used to control the morphology of calcium carbonate crystals. The obtained data showed that the turbidity measurements can be useful in monitoring the kinetics of calcium carbonate crystal growth. The results indicated that in the presence of lysozyme, the shape, and morphology of the crystals were the same as those in the sample without the proteins. The addition of ovalbumin affected the shape of the crystals leading to the formation of a ‘stack-like’ structure. This structure was composed of calcium carbonate and proteins, which was confirmed by FTIR and SEM analysis. The presence of the ovalbumin-lysozyme mixture resulted in the formation of a different structure (i.e., agglomerates of CaCO_3_ and matrix of proteins) compared to the sample with an individual protein. The calcium carbonate that precipitated in the presence of lysozyme, ovalbumin, and the protein mixture was calcite. However, when 0.1% of ovalbumin and the protein mixture were used, a small amount of vaterite appeared after 10 min of precipitation. It was also concluded that, in the presence of the protein mixture, ovalbumin has a critical impact on the calcium carbonate formation, but lysozyme has a negligible influence. This is the result of ovalbumin and calcium ions’ strong interaction, which contributes to the coalescence and formation of a steric barrier preventing particle growth.

## Figures and Tables

**Table 1:** The band assignment of the FTIR vibrations of the calcium carbonate particles precipitated in the absence and presence of proteins [4,10,39,40,41].

Wavenumber [cm^−1^]	Assignment
~713	In-plane deformation mode of CO_3_^2−^ in calcite
~745	In-plane deformation mode of CO_3_^2−^ in vaterite
~876	Out-of-plane deformation mode of CO_3_^2−^
~1085	Symmetric C–O stretching mode
~1420	Asymmetric C–O stretching mode
~1640	C=O stretching mode in I amide
